# Supplementary figures and images for: Deterministic response strategies in a trial-and-error learning task
Source: PLoS Comput Biol. 2018 Nov 29;14(11):e1006621. doi: 10.1371/journal.pcbi.1006621 (PMC6289466; doi:10.1371/journal.pcbi.1006621)

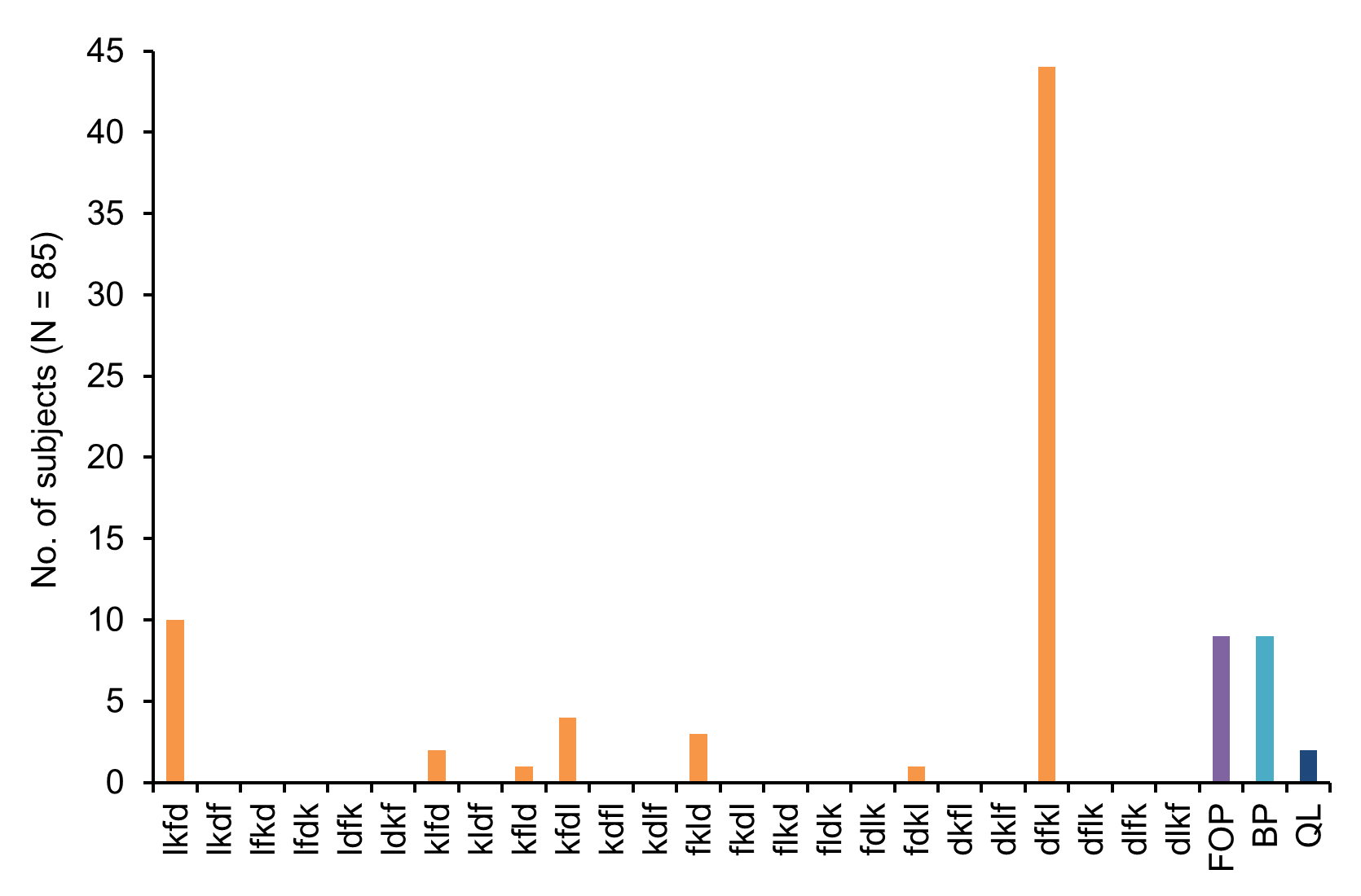

Supplement: S1 Fig — For each subject, it was determined which of the 27 models provided the largest log-likelihood score based on response data of the initial learning phase. Most subjects were best fitted either by the DRP dfkl, DRP lkfd, FOP or BP models. The response orders dfkl and lkfd correspond, respectively, to going from left to right and from right to left on the computer keyboard, which seem to be reasonable response strategies from a human perspective (while from a theoretical perspective, all 24 response orders are equivalent). In contrast, the third-ranked DRP response order kfdl corresponds to the rather implausible sequence right index finger, left index finger, left middle finger, right middle finger, and the fourth-ranked response order fkld corresponds also to an implausible sequence (left index finger, right index finger, right middle finger, left middle finger). Note that the preliminary model comparison reported here is based on the best-ranked model for each subject, with the difference between the best and second best model potentially being arbitrarily small. Thus, the fact that for a few subjects some implausible response orders obtained the highest log-likelihood score seems to reflect a bias in the model comparison procedure: Simply by submitting a larger number of models from the same class to the model comparison procedure, it becomes more likely that a model of this class obtains the highest score. To remove this bias from subsequent analyses, we constrained the model space to the five models DRP dfkl, DRP lkfd, FOP, BP and Q-learning, and conducted statistical tests for model comparison, reported in the main text. (TIF) [file pcbi.1006621.s001.tif]

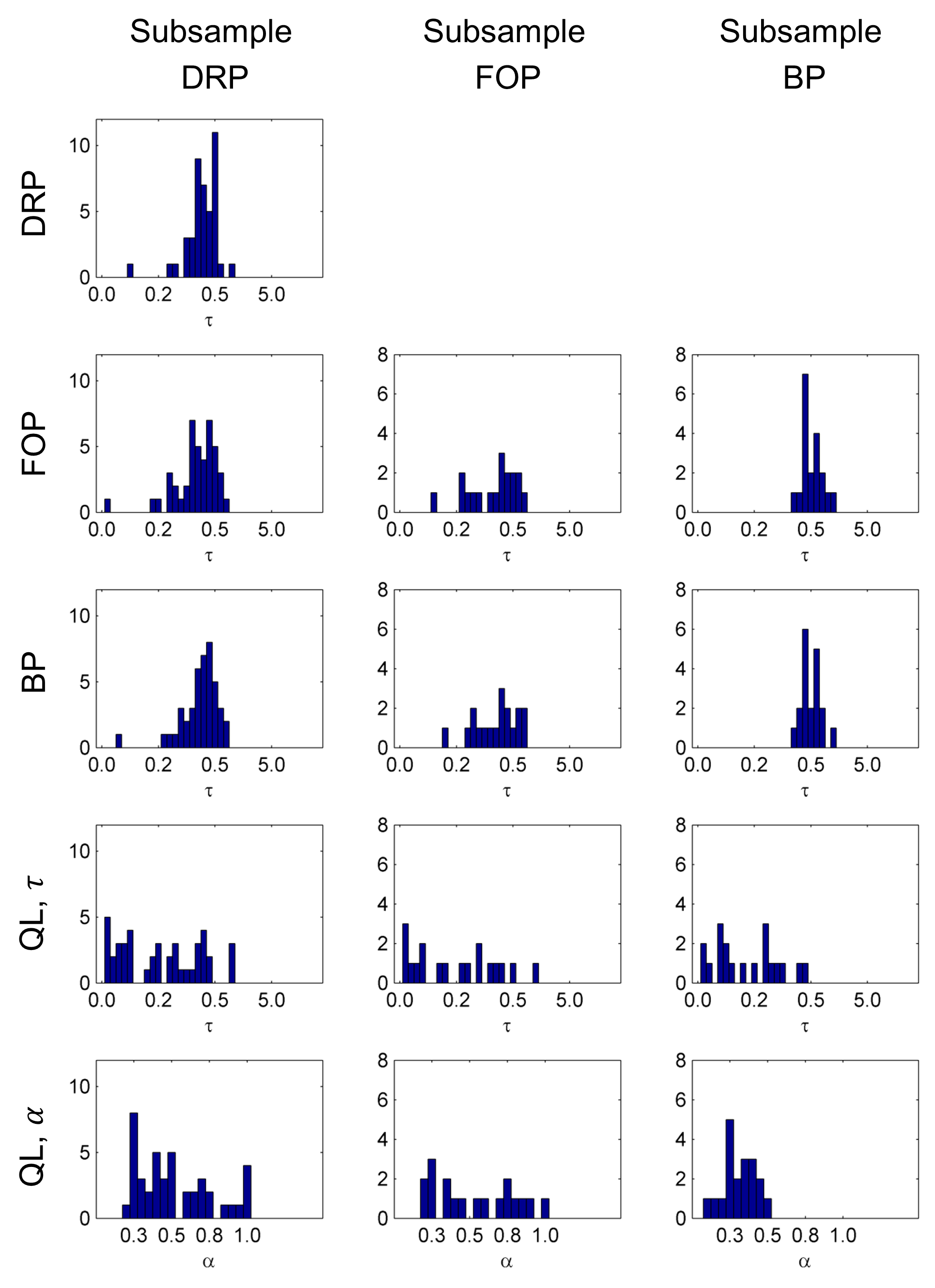

Supplement: S2 Fig — Response selection noise τ was fitted for all four models DRP, FOP, BP and Q-learning, while the learning rate α was only included in the Q-learning model. Response selection noise τ was optimized along the range 0, 1/6, 1/5.8, …, 1/0.2 (31 values), and the learning rate α was selected from the range 0.05, 0.10, …, 1.0 (20 values). Parameters were fitted separately for each subject on response data of the initial learning phase. (TIF) [file pcbi.1006621.s002.tif]

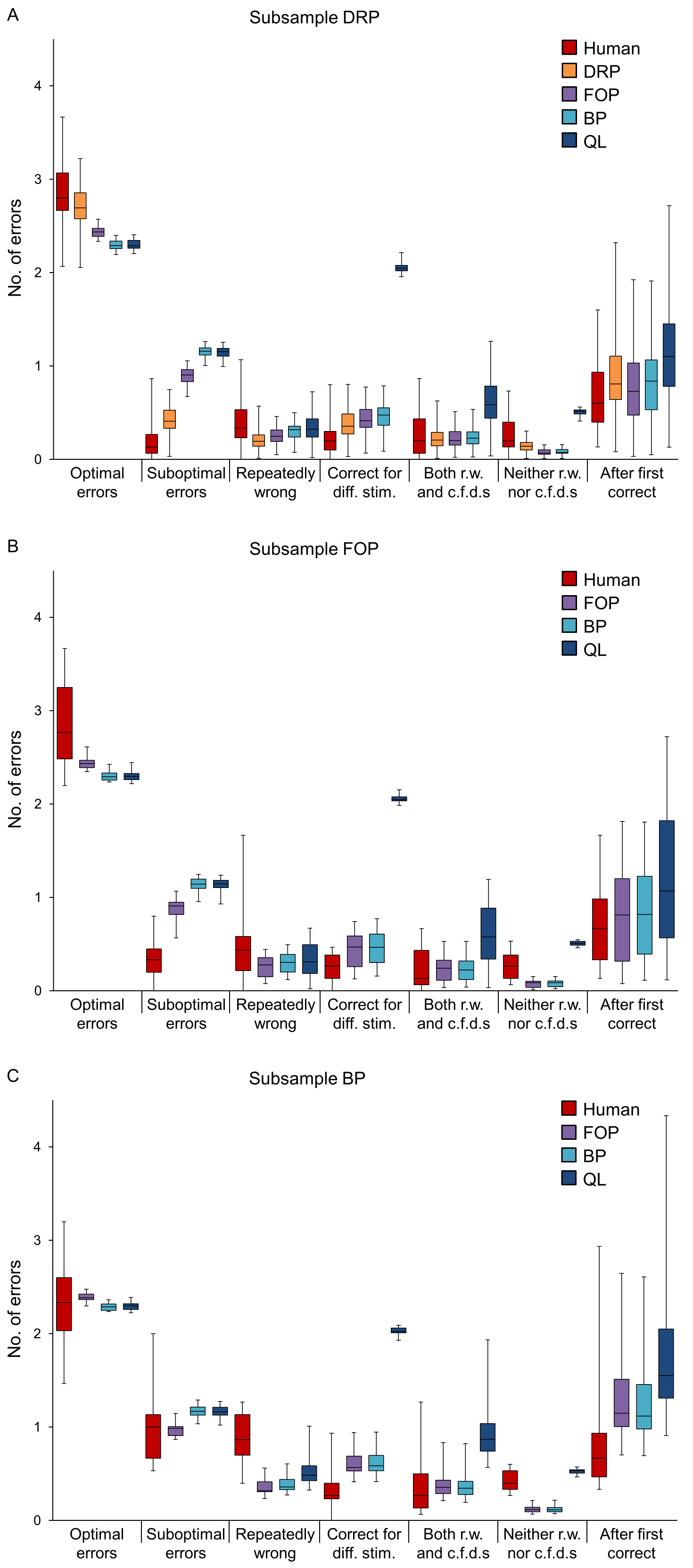

Supplement: S3 Fig — Errors were categorized into 7 different types. Optimal errors were defined as errors that occurred when a response with maximum probability of being correct was selected. Suboptimal errors were defined as errors that occurred when a response with nonzero probability, but not maximal probability, was selected. Errors were categorized as ‘repeatedly wrong’ if negative feedback had been received before for the respective S-R pair. Errors were categorized as ‘correct for a different stimulus’ if a response was selected that had been assigned to a different stimulus in earlier trials. Errors were categorized as ‘both repeatedly wrong and correct for a different stimulus’ if both criteria were fulfilled. Errors were categorized as ‘neither repeatedly wrong nor correct for a different stimulus’ if an indirect inference would have led to the correct response, as for example in step 5 of Fig 3, where the correct response for the fourth stimulus was inferred based on the one-to-one property of the S-R mappings. Finally, errors were categorized as ‘after first correct’ if the respective S-R pair had been performed correctly before. For each subject, the number of errors of each type was averaged across the 15 blocks of interest. The plots show median, first- and third quartile, and minimum and maximum values across the subjects of the respective sample. A: Data of the DRP subsample. As also depicted in Fig 6 of the main text, the DRP models performed considerably better than the other models in terms of optimal and suboptimal errors. The FOP model showed at least a tendency towards the actual data for these two error types, but all three models (FOP, BP and Q-learning) failed to reproduce the high variability of optimal and suboptimal errors found in the actual data. Moreover, the Q-learning model was unable to exploit the one-to-one property of the S-R mappings, as can be seen by the high rate of ‘correct for a different stimulus’, ‘both repeatedly wrong and correct for a di [file pcbi.1006621.s003.tif]

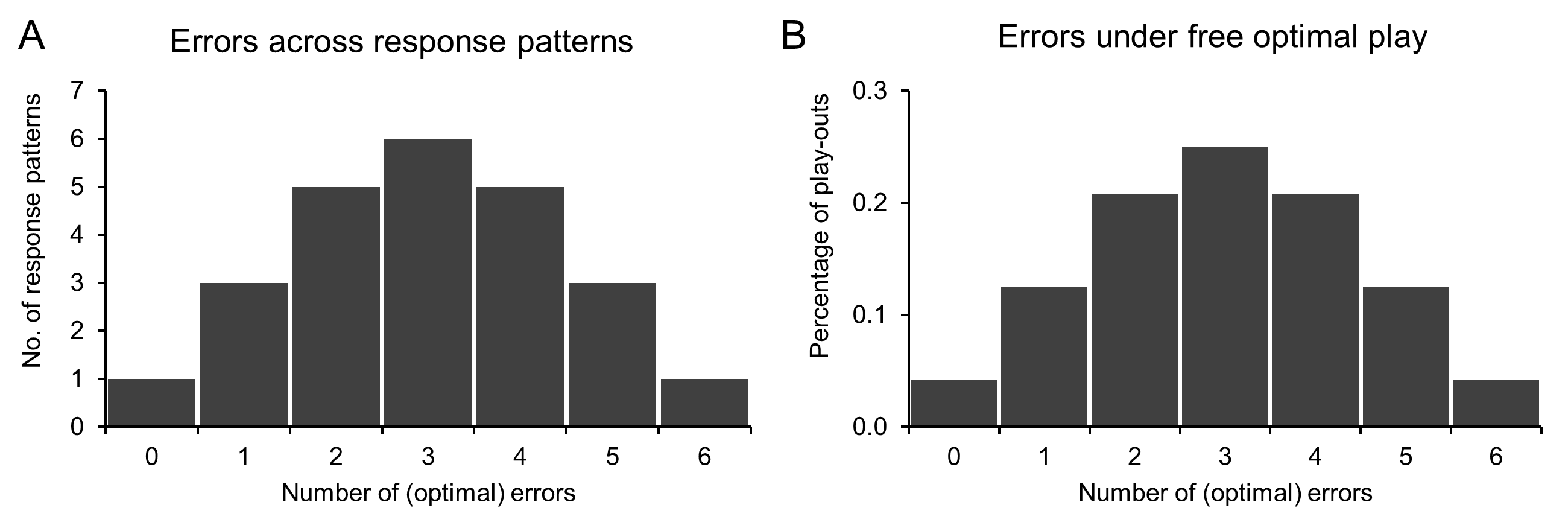

Supplement: S4 Fig — A: For any stimulus sequence, the number of optimal errors produced by the 24 response orders invariably resulted in the shown distribution. B: For a large number of repetitions, the number of errors under free optimal play converged to the same distribution as in A on any stimulus sequence. (TIF) [file pcbi.1006621.s004.tif]

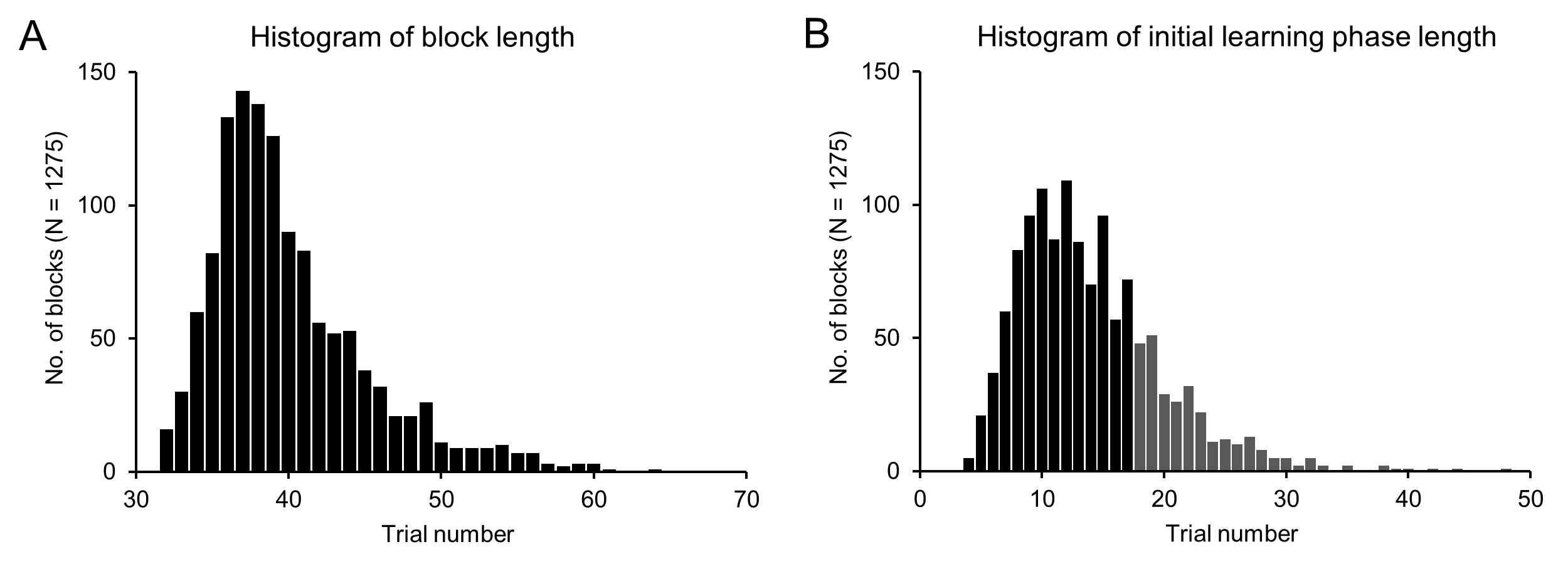

Supplement: S5 Fig — A: Histogram of the overall block length, including blocks 6 to 20 from all subjects (N = 85). The maximum of 70 trials was never reached after block 5. B: Histogram of the initial learning phase length for blocks 6 to 20. The third quartile (trial no. 17) was taken as cut-off in Fig 5A, indicated by the black/gray shading. (TIF) [file pcbi.1006621.s005.tif]
