# Supplementary material for: Deterministic response strategies in a trial-and-error learning task
Source: PLoS Comput Biol. 2018 Nov 29;14(11):e1006621. doi: 10.1371/journal.pcbi.1006621 (PMC6289466; doi:10.1371/journal.pcbi.1006621)
Supplement: S1 Text — (PDF) [file pcbi.1006621.s006.pdf]

Task instructions

Subjects were instructed on the experimental task before starting with the experiment. The instruction text (originally in German) was:

In the following experiment, your task is to learn via trial-and-error how to respond with four fingers to four abstract geometric shapes. In each trial, you will be presented with one of the four shapes. Additionally, four boxes will represent the four response options, outer left box: left middle finger [D], inner left box: left index finger [F], inner right box: right index finger [K], outer right box: right middle finger [L]. If you choose the correct response, you will hear a sound. Elsewise, you will get an error message. The learning task is completed once you have responded correctly eight times to each of the four shapes. To reach this criterion, you have maximally 70 trials available. After completion, you will get feedback on your average error rate, where a value larger than 25% will indicate that you were above chance level. You should also take into account that it is practically impossible to achieve a value of 100%, as at the beginning you will necessarily have to try out some erroneous must-guess responses. During the experiment, you are asked to perform 20 different learning blocks, each with novel abstract shapes and novel sounds following your responses.
