# Supplementary material for: Deterministic response strategies in a trial-and-error learning task
Source: PLoS Comput Biol. 2018 Nov 29;14(11):e1006621. doi: 10.1371/journal.pcbi.1006621 (PMC6289466; doi:10.1371/journal.pcbi.1006621)
Supplement: S2 Text — (PDF) [file pcbi.1006621.s007.pdf]

Group-level BIC scores and log-likelihood values

Based on the request of the reviewers, we aggregated the log-likelihood values across all N = 85 subjects. As in the main text, log-likelihood values were computed based on data from the initial learning phase of learning blocks 6 to 20. Log-likelihood values of each subject were either summed up or averaged before entered into group-level analysis. BIC scores were computed for each subject as

$$\mathrm{BIC} = \ln(n)\, k - 2 \ln \hat{L}$$

where n is the number of trials, k the number of model parameters and $\ln \hat{L}$ the summed up log-likelihood of the data given the model using maximum likelihood model parameters. For all DRP models, as well as the FOP and BP models, the number of model parameters was set to $k = 1$, corresponding to the response selection noise parameter τ. For the Q-learning model, the number of model parameters was set to $k = 2$, corresponding to the response selection noise parameter τ and the learning rate parameter α. Lower BIC scores indicate better model fits.

Moreover, we entered the BIC scores, as well as sums and mean values of log-likelihoods into the *VBA_groupBMC.m* function of the VBA toolbox (Daunizeau J., Adam V., Rigoux L., PLoS Comp. Bio. 2014. VBA: A Probabilistic Treatment of Nonlinear Models for Neurobiological and Behavioural Data) to run random effects group level analyses.

In contrast to the analysis approach presented in the main text, where models were compared separately for each subject, the group level analyses are based on data of all N = 85 subjects. Specifically, in these group level analyses, data from different subsamples (as defined in the main text) are aggregated. The resulting plots are difficult to interpret, as for example the group level evidence for the DRP *lkfd* model is based on the log-likelihood values of the 7 subjects who implemented this learning scheme but also on the log-likelihood values of the 78 subjects who showed low evidence for this procedure. The group-level comparisons result in lower evidence for the DRP lkfd model than the Q-learning model, which however, according to the main text analysis, did not provide a good fit for any individual subject for the initial learning phase.

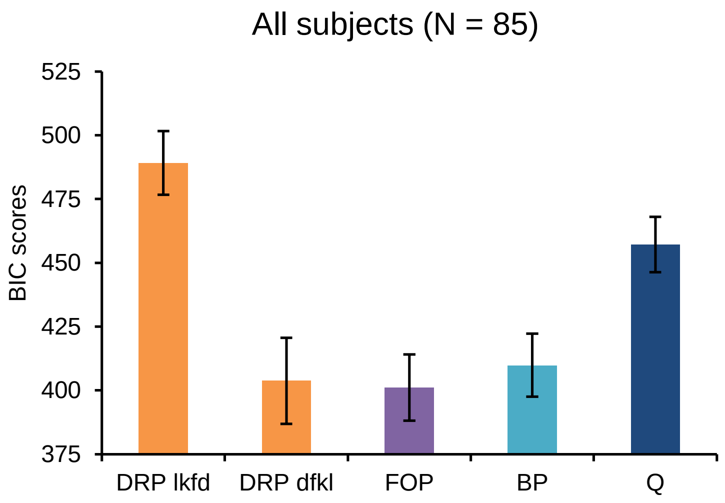

S2 Text, Figure 1. Group-level mean values of BIC scores including all N = 85 subjects. Error bars indicate SEM.

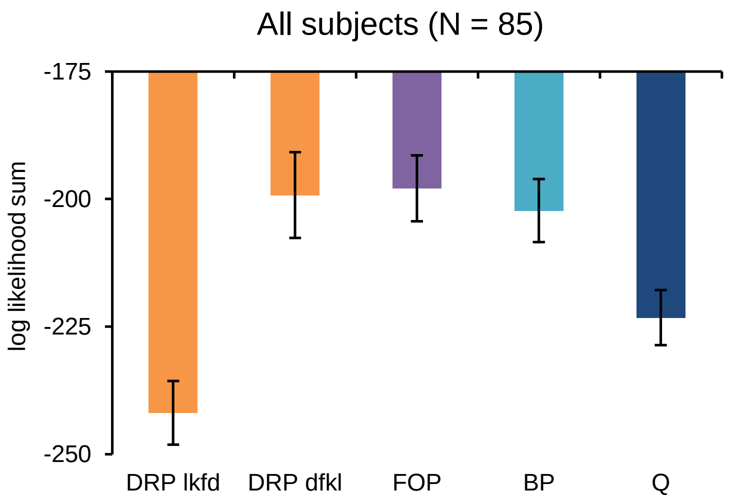

S2 Text, Figure 2. Log-likelihood values, averaged across all N = 85 subjects. Before entered into group-level analysis, log-likelihood values were summed up across trials for each subject. Error bars indicate SEM.

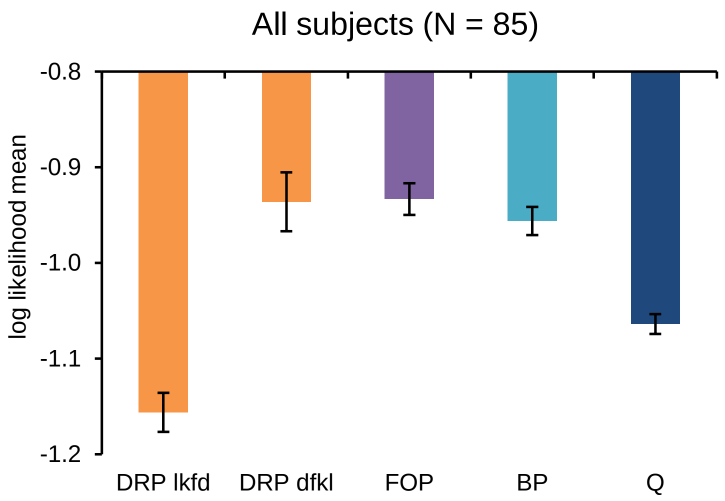

S2 Text, Figure 3. Log-likelihood values, averaged across all N = 85 subjects. Before entered into group-level analysis, log-likelihood values were averaged across trials for each subject. Error bars indicate SEM.

Output from *VBA_groupBMC.m* using BIC scores

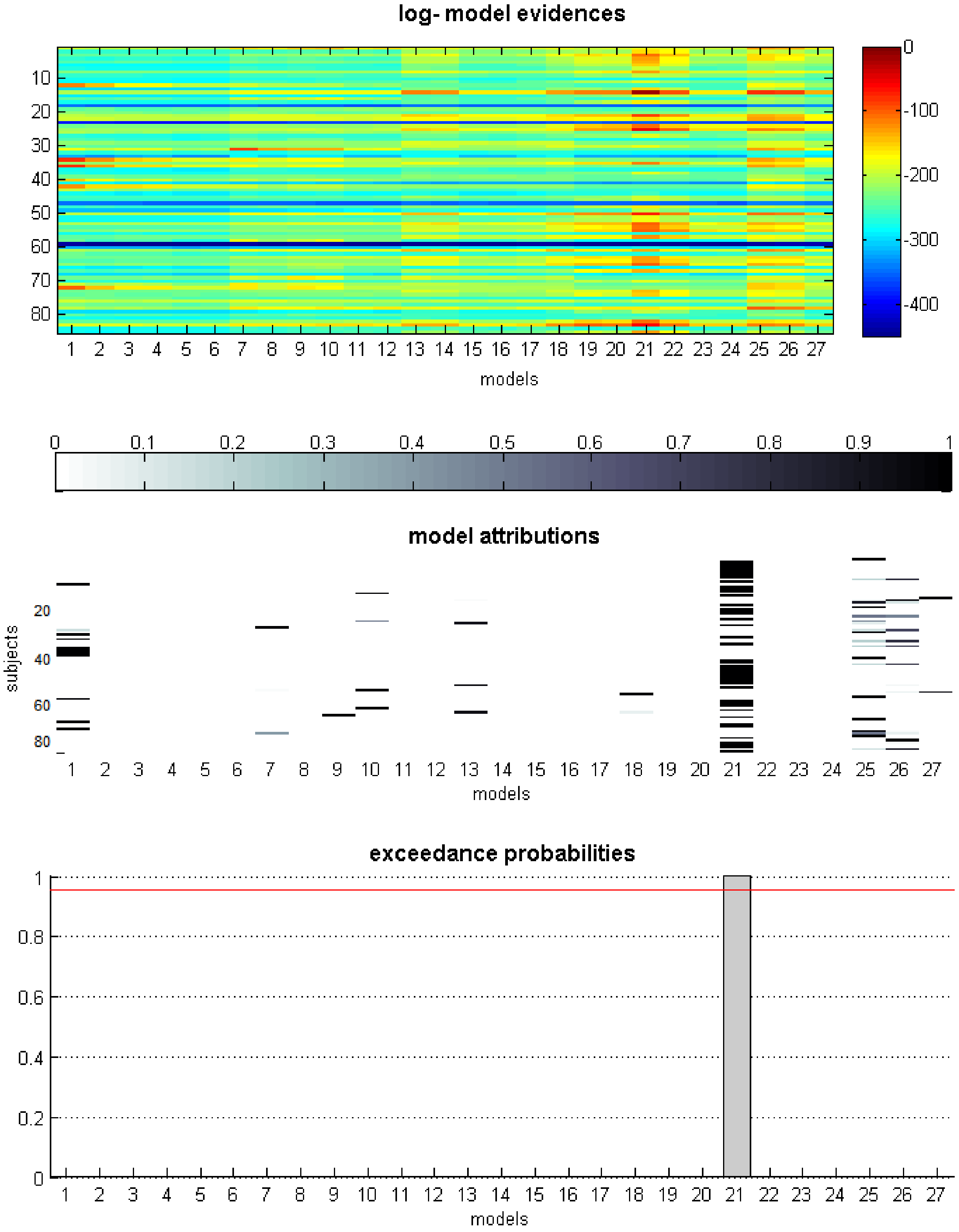

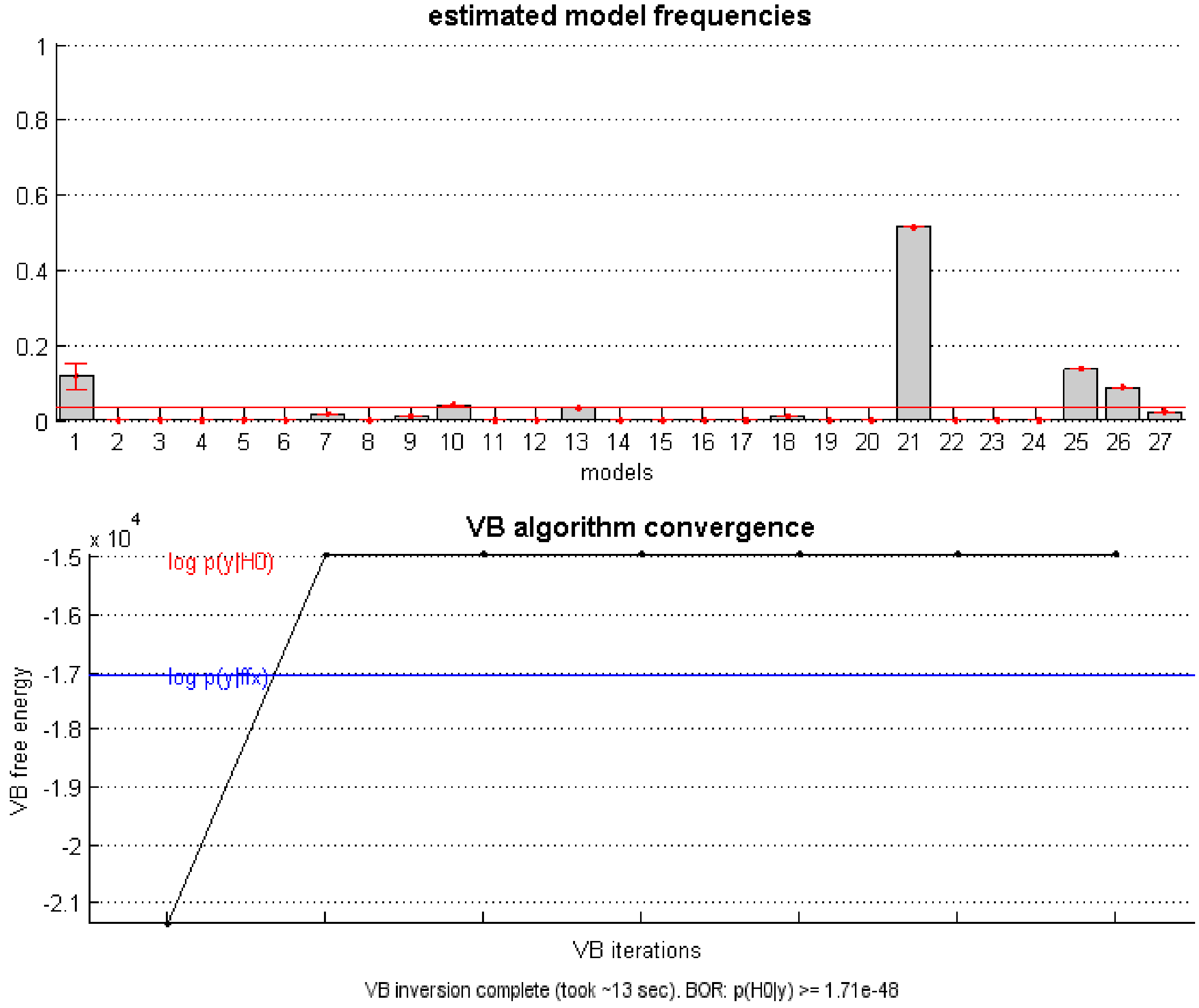

S2 Text, Figure 4. Results of the RFX group-level analysis using the VBA toolbox. BIC scores were transformed into -0.5*BIC before being entered into the analysis. Model numbers as in Figure S1: 1 = DRP *lkfd*, 21 = DRP *dfkl*, 25 = FOP, 26 = BP, 27 = QL.

Output from *VBA_groupBMC.m* using summed log-likelihood values

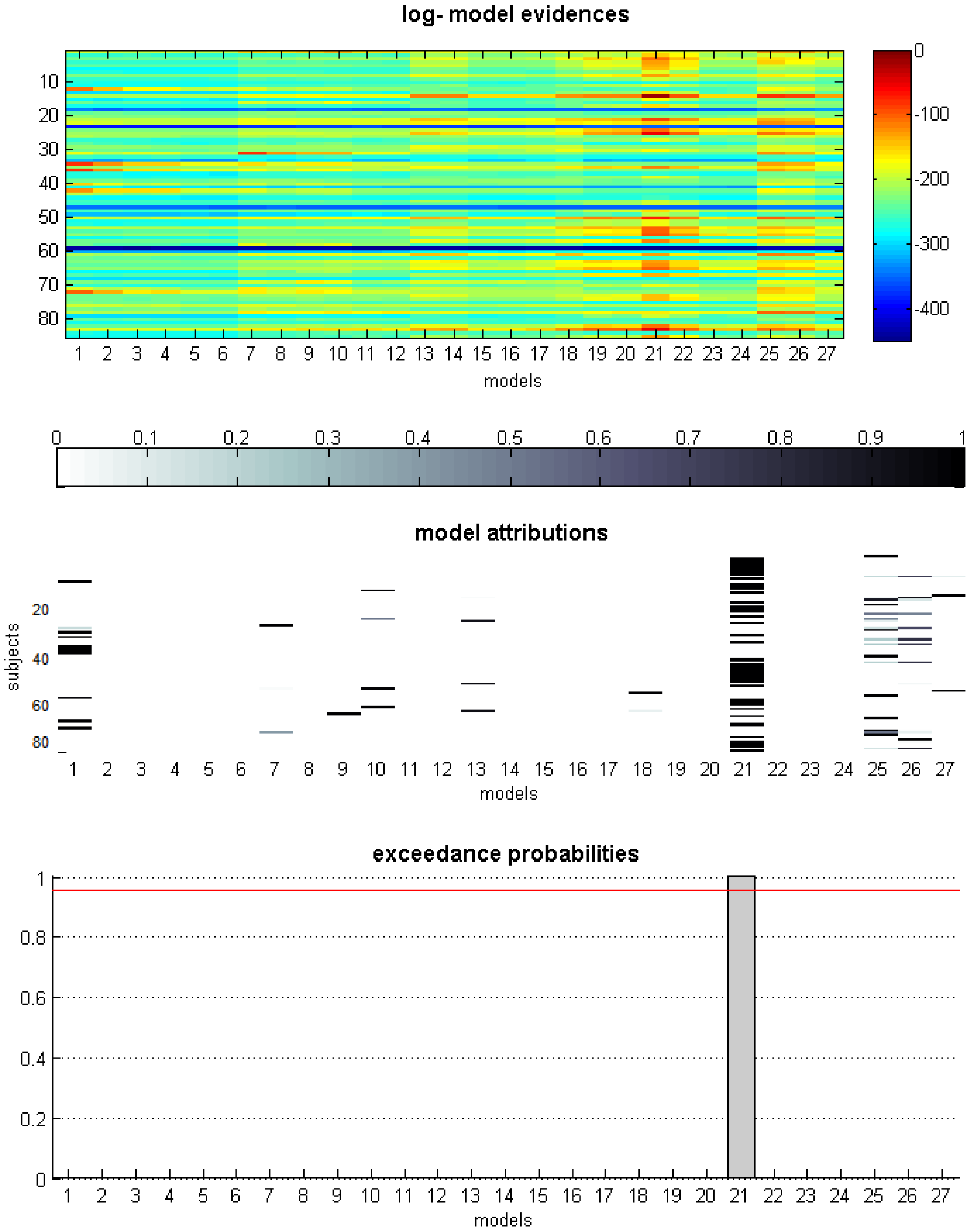

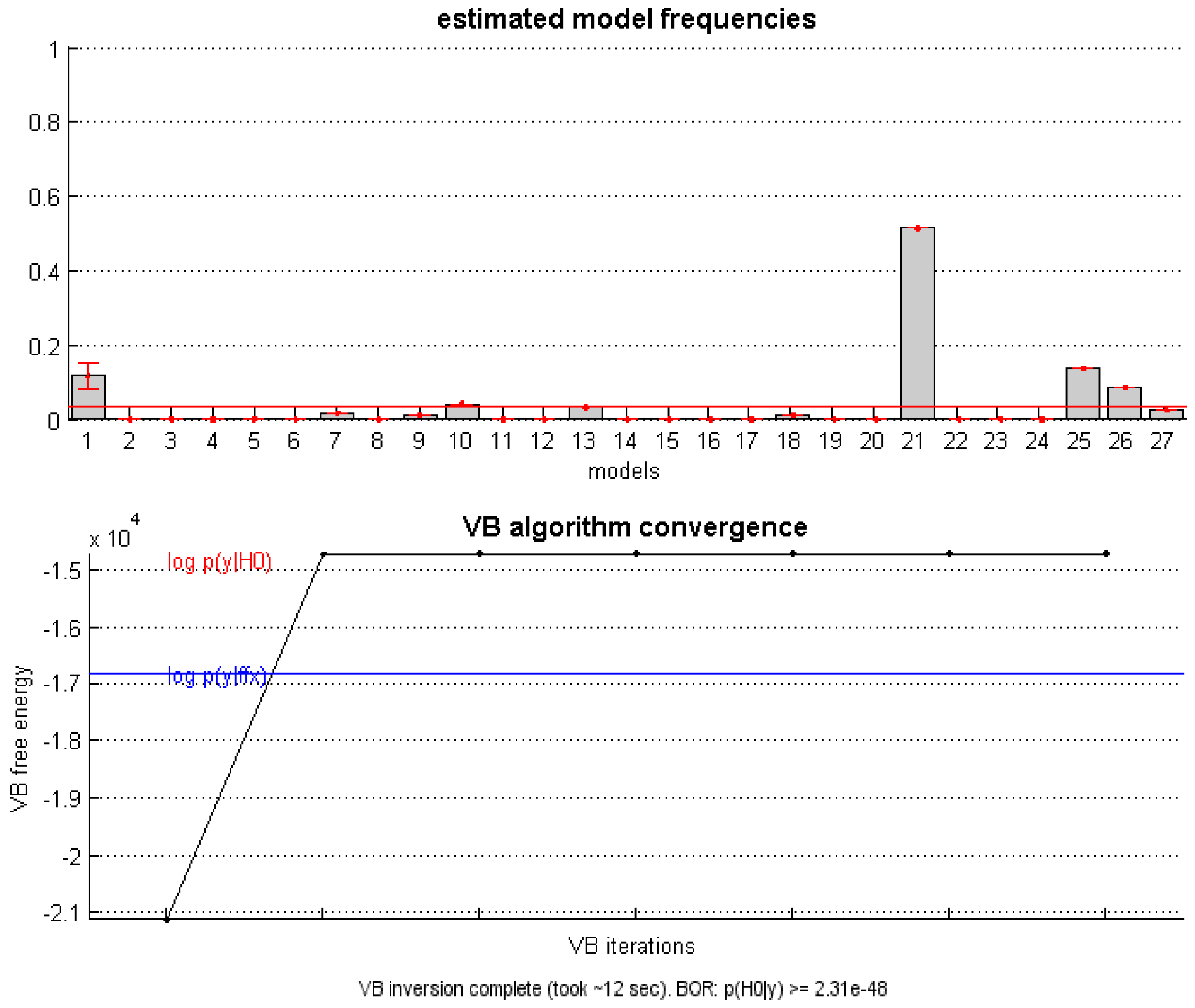

S2 Text, Figure 5. Results of the RFX group-level analysis using the VBA toolbox. Log-likelihood values were summed up across trials for each subject before entered into the group-level analysis. Model numbers as in Figure S1: 1 = DRP *lkfd*, 21 = DRP *dfkl*, 25 = FOP, 26 = BP, 27 = QL.

Output from *VBA_groupBMC.m* using averaged log-likelihood values

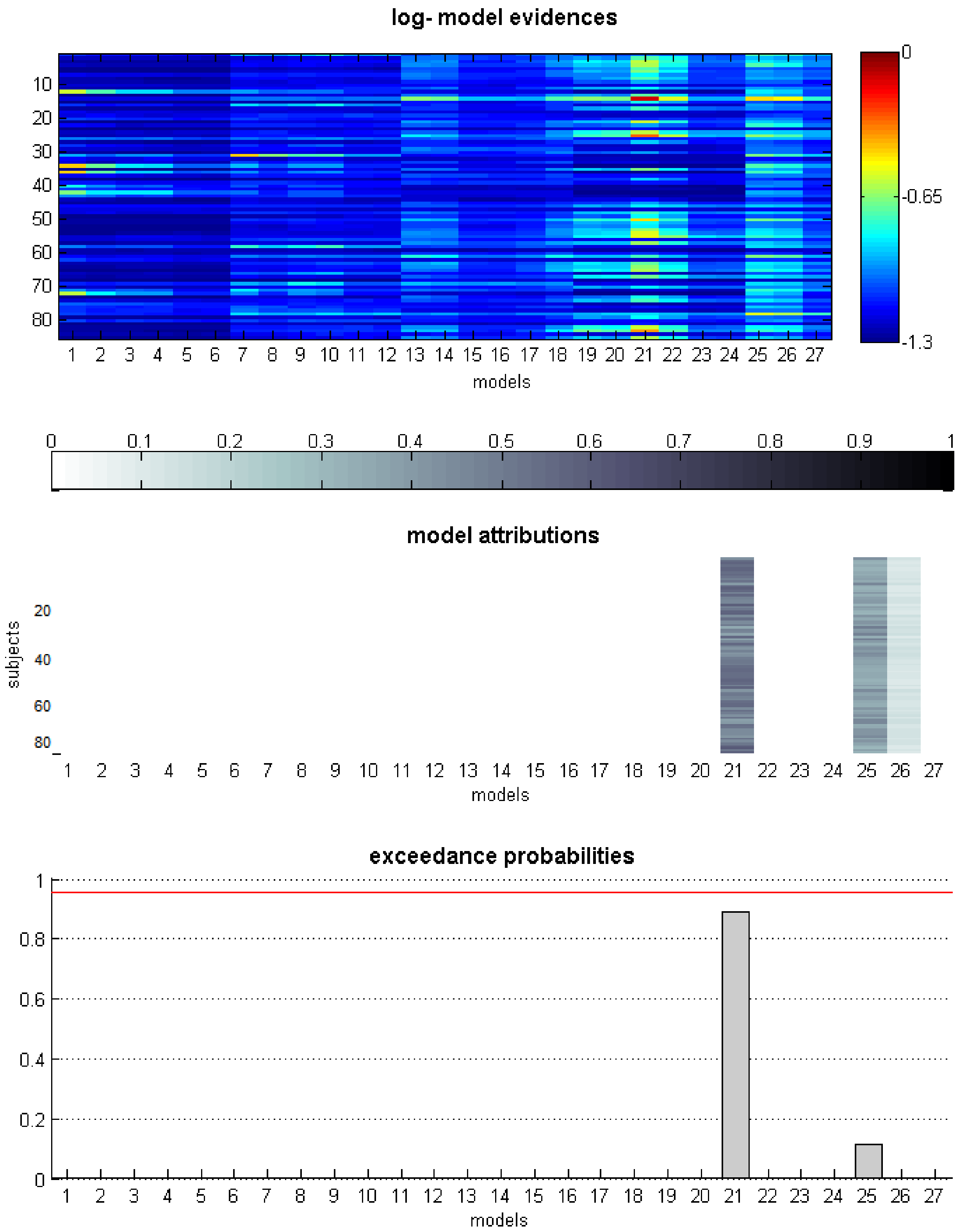

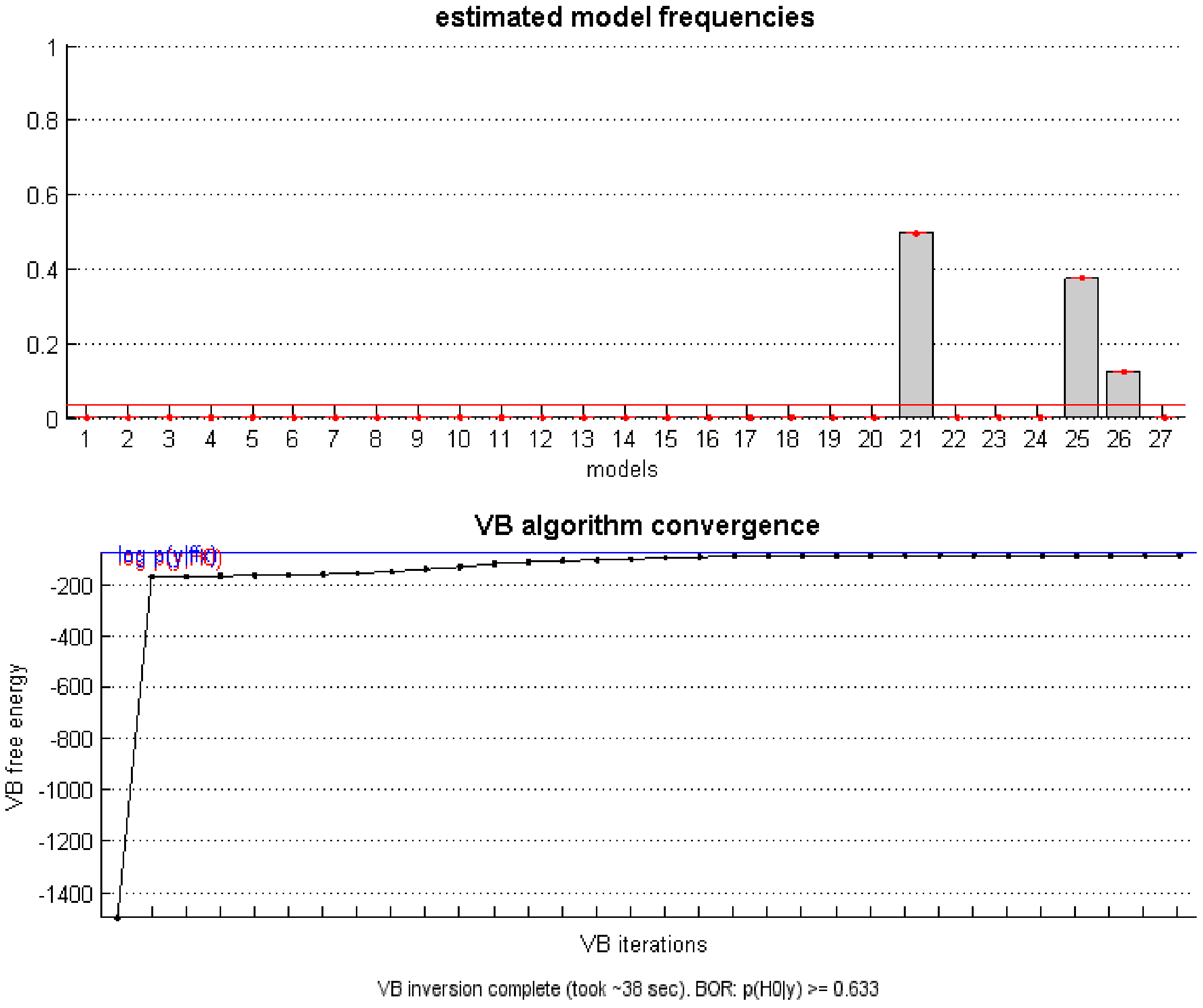

S2 Text, Figure 6. Results of the RFX group-level analysis using the VBA toolbox. Log-likelihood values were averaged across trials for each subject before entered into the group-level analysis. Model numbers as in Figure S1: 1 = DRP *lkfd*, 21 = DRP *dfkl*, 25 = FOP, 26 = BP, 27 = QL.
