# Supplementary material for: Deterministic response strategies in a trial-and-error learning task
Source: PLoS Comput Biol. 2018 Nov 29;14(11):e1006621. doi: 10.1371/journal.pcbi.1006621 (PMC6289466; doi:10.1371/journal.pcbi.1006621)
Supplement: S1 Appendix — (PDF) [file pcbi.1006621.s008.pdf]

Appendix

Notation. Experimental trials are indexed with $t = 1, 2, 3, \ldots$, and each trial consists of a stimulus $s_t \in \{1, 2, 3, 4\}$, a response $r_t \in \{d, f, k, l\}$ and an outcome $o_t \in \{0, 1\}$. After completion of trial no. t, the history of stimuli, responses and outcomes is denoted as $H_t = \{(s)_t, (r)_t, (o)_t\}$, with $(s)_t = (s_1, s_2, \ldots, s_t)$, $(r)_t = (r_1, r_2, \ldots, r_t)$, $(o)_t = (o_1, o_2, \ldots, o_t)$.
Let $\{M_i | i = 1, 2, 3, \ldots, 24\}$ be the set of the 24 one-to-one mappings between four stimuli and four responses as shown in Figure 2 in the main text, with deterministic outcomes being either zero or one. In a probabilistic framework, the one-to-one property of S-R mappings with deterministic outcomes translates into: For a given mapping M_i, for each stimulus $\hat{s}$, there exists a response $\hat{r}$ such that $P[o = 1 | M_i, \hat{s}, \hat{r}] = 1$, and $P[o = 0 | M_i, \hat{s}, r] = 1$ for all responses $r \neq \hat{r}$ and $P[o = 0 | M_i, s, \hat{r}] = 1$ for all stimuli $s \neq \hat{s}$.
We will show that in order to maximize expected reward and minimize expected uncertainty, it is sufficient to track the probability distribution over the S-R mappings $M_i, i = 1, 2, \ldots, 24$ given the history of S-R-O combinations, that is, it is sufficient to track $P[M_i | H_t]$ for all $i = 1, 2, \ldots, 24$. Initially, without any S-R-O information available, we assume that all mappings are equally likely, that is, we define $P[M_i | H_0] = 1/24$ for all $i = 1, 2, \ldots, 24$.

Computation of outcome probabilities. First, we show that when stimulus s_t is presented, the likelihood for getting an outcome o_t by responding with r_t can be computed as:

$$P[o_t | s_t, r_t, H_{t-1}] = \sum_{i=1}^{24} P[o_t | M_i, s_t, r_t] P[M_i | H_{t-1}] \qquad \textbf{(1)}$$

It is:

$$P[o_t | s_t, r_t, H_{t-1}]$$

$$= P[o_t | s_t, r_t, H_{t-1}] \sum_{i=1}^{24} P[M_i | H_t]$$

$$= \sum_{i=1}^{24} P[M_i | o_t, s_t, r_t, H_{t-1}] P[o_t | s_t, r_t, H_{t-1}]$$

As generally $P[A|B,C]P[B|C] = P[B|A,C]P[A|C]$, it follows with $A = M_i$, $B = o_t$ and $C = \{s_t, r_t, H_{t-1}\}$ that

$$\cdots = \sum_{i=1}^{24} P[o_t | M_i, s_t, r_t, H_{t-1}] P[M_i | s_t, r_t, H_{t-1}]$$

$$= \sum_{i=1}^{24} P[o_t | M_i, s_t, r_t] P[M_i | H_{t-1}]$$

Maximization of expected reward. As $P[o_t | M_i, s_t, r_t]$ is known by the definition of M_i, it is sufficient to have $P[M_i | H_{t-1}]$ at hand in order to maximize expected reward in trial t:

$$\max_{r_t} E[o_t | s_t, r_t, H_{t-1}]$$

$$= \max_{r_t} 1 \cdot P[o_t = 1 | s_t, r_t, H_{t-1}] + 0 \cdot P[o_t = 0 | s_t, r_t, H_{t-1}]$$

$$\overset{(1)}{=} \max_{r_t} \sum_{i=1}^{24} P[o_t = 1 | M_i, s_t, r_t] P[M_i | H_{t-1}]$$

Updating. After having obtained an outcome o_t for response r_t, the likelihood for each mapping M_i can be updated in the following way:

$$P[M_i|H_t] = \frac{P[o_t|M_i,s_t,r_t]P[M_i|H_{t-1}]}{\sum_{j=1}^{24} P[o_t|M_j,s_t,r_t]P[M_j|H_{t-1}]} \quad \textbf{(2)}$$

From this identity, we can see that by starting with a uniform distribution, the distribution over the M_i's (i.e. $P[M.|H_t]$) evolves as a mixture of a uniform distribution over a subset of M_i's and zeros for the rest of the M_i's, that is, after trial t exists an $n_t \in \{1,2,\ldots,24\}$ and subindexes $\{i_1,i_2,\ldots,i_{n_t}\}$, such that $P[M_i|H_t] = 1/n_t$ for all $i \in \{i_1,i_2,\ldots,i_{n_t}\}$ and $P[M_i|H_t] = 0$ for all $i \in \{1,2,\ldots,24\} \setminus \{i_1,i_2,\ldots,i_{n_t}\}$. To this end, we assume the properties hold for the previous trial, i.e. it is $P[M_i|H_{t-1}] \in \{0,1/n_{t-1}\}$ for all $i = 1,2,\ldots,24$. Generally, it is $P[o_t|M_i,s_t,r_t] \in \{0,1\}$. If follows directly from (2) that $P[M_i|H_t] = 0$ if $P[M_i|H_{t-1}] = 0$ or $P[o_t|M_i,s_t,r_t] = 0$. Hence, we assume that $P[M_i|H_{t-1}] = 1/n_{t-1}$ and $P[o_t|M_i,s_t,r_t] = 1$. We define $n_t = \#\{j \in \{1,2,\ldots,24\}|P[o_t|M_j,s_t,r_t] = 1, P[M_j|H_{t-1}] = 1/n_{t-1}\}$., Then, using (2) again, it is $P[M_i|H_t] = \frac{1/n_{t-1}}{n_t/n_{t-1}} = 1/n_t$. Thus, for all $i \in \{1,2,\ldots,24\}$, it is $P[M_i|H_t] \in \{0,1/n_t\}$.

To equation (2): Generally, it is

$$P[A|B,C] = \frac{P[B|A,C]P[A|C]}{P[B|C]}$$

With $A = M_i$, $B = o_t$ and $C = \{s_t,r_t,H_{t-1}\}$ it follows that

$$P[M_i|H_t]$$

$$= P[M_i|o_t,s_t,r_t,H_{t-1}]$$

$$= \frac{P[o_t|M_i,s_t,r_t,H_{t-1}]P[M_i|s_t,r_t,H_{t-1}]}{P[o_t|s_t,r_t,H_{t-1}]}$$

$$\overset{(1)}{=} \frac{P[o_t|M_i,s_t,r_t]P[M_i|H_{t-1}]}{\sum_{j=1}^{24} P[o_t|M_j,s_t,r_t]P[M_j|H_{t-1}]}$$

Minimization of expected uncertainty. Next, we show that maximizing expected reward in the current trial also minimizes the expected uncertainty. To this end, at the beginning of trial t, we define the set of S-R mappings that are consistent with the S-R-0 history: $\mathcal{C}_{t-1} = \{i \in \{1,2,\ldots,24\}|P[M_i|H_{t-1}] > 0\}$ and $n_{t-1} = \#\mathcal{C}_{t-1}$. Moreover, given the currently presented stimulus s_t, we define for the four responses d, f, k, l the sets of S-R mappings that are consistent with a positive outcome:

$$\mathcal{C}_t^d = \{i \in \{1,2,\ldots,24\}|i \in \mathcal{C}_{t-1}, P[o_t = 1|M_i,s_t,r_t = d] = 1\}$$

$$\mathcal{C}_t^f = \{i \in \{1,2,\ldots,24\}|i \in \mathcal{C}_{t-1}, P[o_t = 1|M_i,s_t,r_t = f] = 1\}$$

$$\mathcal{C}_t^k = \{i \in \{1,2,\ldots,24\}|i \in \mathcal{C}_{t-1}, P[o_t = 1|M_i,s_t,r_t = k] = 1\}$$

$$\mathcal{C}_t^l = \{i \in \{1,2,\ldots,24\}|i \in \mathcal{C}_{t-1}, P[o_t = 1|M_i,s_t,r_t = l] = 1\}$$

And accordingly, we define $n_t^d = \#\mathcal{C}_t^d$, $n_t^f = \#\mathcal{C}_t^f$, $n_t^k = \#\mathcal{C}_t^k$, $n_t^l = \#\mathcal{C}_t^l$.

From equation (1) follows, with response $x \in \{d, f, k, l\}$:

$$P[o_t = 1|s_t, r_t = x, H_{t-1}] = \sum_{i=1}^{24} P[o_t = 1|M_i, s_t, r_t = x] P[M_i|H_{t-1}]$$

Moreover, as we have seen above, S-R mappings in $\mathcal{C}_{t-1}$ are equally likely given H_{t-1} (i.e. $P[M_i|H_{t-1}] = 1/n_{t-1}$ for all $i \in \mathcal{C}_{t-1}$), hence we get for $x \in \{d, f, k, l\}$:

$$P[o_t = 1|s_t, r_t = x, H_{t-1}] = \frac{n_t^x}{n_{t-1}}$$

Additionally, from the one-to-one property of the S-R mappings follows that

$$n_t^d + n_t^f + n_t^k + n_t^l = n_{t-1}$$

Importantly, the updating procedure from equation (2) implies that when response x (with $x \in \{d, f, k, l\}$) is given (i.e. $r_t = x$) and followed by a positive outcome (i.e. $o_t = 1$), then $P[M_i|H_t] = 1/n_t^x$ for $i \in \mathcal{C}_t^x$ and $P[M_i|H_t] = 0$ for $i \in \{1, 2, \ldots, 24\} \setminus \mathcal{C}_t^x$, whereas if the response is not rewarded ($o_t = 0$), then $P[M_i|H_t] = 1/(n_{t-1} - n_t^x)$ for $i \in \mathcal{C}_{t-1} \setminus \mathcal{C}_t^x$ and $P[M_i|H_t] = 0$ for $i \in \{1, 2, \ldots, 24\} \setminus (\mathcal{C}_{t-1} \setminus \mathcal{C}_t^x)$.

Without loss of generality, we assume that response d maximizes expected reward:

$$P[o_t = 1|s_t, r_t = d, H_{t-1}] = \max_{x \in \{d,f,k,l\}} P[o_t = 1|s_t, r_t = x, H_{t-1}]$$

Equivalently, it is $n_d = \max\{n_d, n_f, n_k, n_l\}$. We want to show that response d also minimizes the expected uncertainty of the S-R mappings, with uncertainty being defined as the entropy H of the distribution $P[M.|H_t]$, i.e. we want to show:

$$E[\mathrm{H}(P[M.|H_t])|s_t, r_t = d, H_{t-1}] = \min_{x \in \{d,f,k,l\}} E[\mathrm{H}(P[M.|H_t])|s_t, r_t = x, H_{t-1}]$$

Given the considerations above, it follows that:

$$E[\mathrm{H}(P[M.|H_t])|s_t, r_t = x, H_{t-1}]$$

$$= E[\mathrm{H}(P[M.|s_t, r_t = x, o_t, H_{t-1}])|s_t, r_t = x, H_{t-1}]$$

$$= E[\mathrm{H}(P[M.|s_t, r_t = x, o_t, H_{t-1}])1_{\{o_t=1\}}|s_t, r_t = x, H_{t-1}]$$
$$+ E[\mathrm{H}(P[M.|s_t, r_t = x, o_t, H_{t-1}])1_{\{o_t=0\}}|s_t, r_t = x, H_{t-1}]$$

$$= \mathrm{H}(P[M.|s_t, r_t = x, o_t = 1, H_{t-1}])P[o_t = 1|s_t, r_t = x, H_{t-1}]$$
$$+ \mathrm{H}(P[M.|s_t, r_t = x, o_t = 0, H_{t-1}])P[o_t = 0|s_t, r_t = x, H_{t-1}]$$

$$= \Big(-\sum_{i=1}^{n_t^x} \frac{1}{n_t^x} \log(\frac{1}{n_t^x})\Big)\frac{n_t^x}{n_{t-1}} + \Big(-\sum_{i=1}^{n_{t-1}-n_t^x} \frac{1}{n_{t-1} - n_t^x} \log(\frac{1}{n_{t-1} - n_t^x})\Big)\frac{n_{t-1} - n_t^x}{n_{t-1}}$$

$$= -\frac{n_t^x}{n_{t-1}} \log(\frac{1}{n_t^x}) - \frac{n_{t-1} - n_t^x}{n_{t-1}} \log(\frac{1}{n_{t-1} - n_t^x})$$

$$= \frac{n_t^x}{n_{t-1}} \log(n_t^x) + \frac{n_{t-1} - n_t^x}{n_{t-1}} \log(n_{t-1} - n_t^x)$$

$$= \frac{1}{n_{t-1}} \log\Big((n_t^x)^{n_t^x}(n_{t-1} - n_t^x)^{n_{t-1}-n_t^x}\Big)$$

We show exemplarily for response f that from $n_d \geq n_f$ follows that

$$E[\mathrm{H}(P[M.|H_t])|s_t, r_t = d, H_{t-1}] \leq E[\mathrm{H}(P[M.|H_t])|s_t, r_t = f, H_{t-1}]$$

Exploiting the transformation from above, we have to show that

$$\frac{1}{n_{t-1}} \log\left((n_t^d)^{n_t^d}(n_{t-1} - n_t^d)^{n_{t-1}-n_t^d}\right) \leq \frac{1}{n_{t-1}} \log\left((n_t^f)^{n_t^f}(n_{t-1} - n_t^f)^{n_{t-1}-n_t^f}\right)$$

Which can be simplified further as follows:

$$(n_t^d)^{n_t^d}(n_{t-1} - n_t^d)^{n_{t-1}-n_t^d} \leq (n_t^f)^{n_t^f}(n_{t-1} - n_t^f)^{n_{t-1}-n_t^f}$$

$$(n_t^d)^{n_t^d}(n_t^f + n_t^k + n_t^l)^{n_t^f+n_t^k+n_t^l} \leq (n_t^f)^{n_t^f}(n_t^d + n_t^k + n_t^l)^{n_t^d+n_t^k+n_t^l}$$

$$(n_t^d)^{n_t^d}(n_t^f + n_t^k + n_t^l)^{n_t^f}(n_t^f + n_t^k + n_t^l)^{n_t^k+n_t^l} \leq (n_t^f)^{n_t^f}(n_t^d + n_t^k + n_t^l)^{n_t^d}(n_t^d + n_t^k + n_t^l)^{n_t^k+n_t^l}$$

If $n_t^f = 0$, the inequality simplifies to

$$(n_t^d)^{n_t^d}(n_t^k + n_t^l)^{n_t^k+n_t^l} \leq (n_t^d + n_t^k + n_t^l)^{n_t^d}(n_t^d + n_t^k + n_t^l)^{n_t^k+n_t^l}$$

which is correct, given that $n_t^d \geq 1$ and $n_t^k, n_t^l \geq 0$.
Hence, we continue with $n_t^f > 0$:

$$\left(\frac{n_t^f + n_t^k + n_t^l}{n_t^f}\right)^{n_t^f} \leq \left(\frac{n_t^d + n_t^k + n_t^l}{n_t^d}\right)^{n_t^d}\left(\frac{n_t^d + n_t^k + n_t^l}{n_t^f + n_t^k + n_t^l}\right)^{n_t^k+n_t^l}$$

From $n_d \geq n_f$ follows that

$$\left(\frac{n_t^d + n_t^k + n_t^l}{n_t^f + n_t^k + n_t^l}\right)^{n_t^k+n_t^l} \geq 1$$

Hence, it remains to show that

$$\left(\frac{n_t^f + n_t^k + n_t^l}{n_t^f}\right)^{n_t^f} \leq \left(\frac{n_t^d + n_t^k + n_t^l}{n_t^d}\right)^{n_t^d}$$

$$1 + \frac{n_t^k + n_t^l}{n_t^f} \leq \left(1 + \frac{n_t^k + n_t^l}{n_t^d}\right)^{\frac{n_t^d}{n_t^f}}$$

$$1 + \frac{n_t^d}{n_t^f} \cdot \frac{n_t^k + n_t^l}{n_t^d} \leq \left(1 + \frac{n_t^k + n_t^l}{n_t^d}\right)^{\frac{n_t^d}{n_t^f}}$$

With $r = n_t^d / n_t^f$ and $x = (n_t^k + n_t^l)/n_t^d$, this is equivalent to Bernoulli's inequality:

$$1 + rx \leq (1 + x)^r$$

Maximization of expected information gain. Finally, a response that maximizes the expected reward also maximizes the expected information gain, with information gain being defined as the Kullback-Leibler divergence between $P[M.|H_t]$ and $P[M.|H_{t-1}]$. As above, we assume without loss of generality that $n_d = \max\{n_d, n_f, n_k, n_l\}$, and exemplarily show for response f that:

$$E[D_{\mathrm{KL}}(P[M.|H_t] \parallel P[M.|H_{t-1}])|s_t, r_t = d, H_{t-1}] \geq E[D_{\mathrm{KL}}(P[M.|H_t] \parallel P[M.|H_{t-1}])|s_t, r_t = f, H_{t-1}]$$

Generally, for response $x \in \{d, f, k, l\}$, it is:

$$E[D_{\mathrm{KL}}(P[M.|H_t] \parallel P[M.|H_{t-1}])|s_t, r_t = x, H_{t-1}]$$

$$= E[D_{\mathrm{KL}}(P[M.|s_t, r_t = x, o_t, H_{t-1}] \parallel P[M.|H_{t-1}])|s_t, r_t = x, H_{t-1}]$$

$$= E[D_{\mathrm{KL}}(P[M.|s_t, r_t = x, o_t, H_{t-1}] \parallel P[M.|H_{t-1}])1_{\{o_t=1\}}|s_t, r_t = x, H_{t-1}]$$
$$+ E[D_{\mathrm{KL}}(P[M.|s_t, r_t = x, o_t, H_{t-1}] \parallel P[M.|H_{t-1}])1_{\{o_t=0\}}|s_t, r_t = x, H_{t-1}]$$

$$= D_{\mathrm{KL}}(P[M.|s_t, r_t = x, o_t = 1, H_{t-1}] \parallel P[M.|H_{t-1}])P[o_t = 1|s_t, r_t = x, H_{t-1}]$$
$$+ D_{\mathrm{KL}}(P[M.|s_t, r_t = x, o_t = 0, H_{t-1}] \parallel P[M.|H_{t-1}])P[o_t = 0|s_t, r_t = x, H_{t-1}]$$

$$= \Big(\sum_{i=1}^{n_t^x} \frac{1}{n_t^x} \log\big(\frac{\frac{1}{n_t^x}}{\frac{1}{n_{t-1}}}\big)\Big)\frac{n_t^x}{n_{t-1}} + \Big(\sum_{i=1}^{n_{t-1}-n_t^x} \frac{1}{n_{t-1}-n_t^x} \log\big(\frac{\frac{1}{n_{t-1}-n_t^x}}{\frac{1}{n_{t-1}}}\big)\Big)\frac{n_{t-1}-n_t^x}{n_{t-1}}$$

$$= \frac{n_t^x}{n_{t-1}} \log\big(\frac{n_{t-1}}{n_t^x}\big) + \frac{n_{t-1}-n_t^x}{n_{t-1}} \log\big(\frac{n_{t-1}}{n_{t-1}-n_t^x}\big)$$

$$= \log(n_{t-1}) + \frac{n_t^x}{n_{t-1}} \log\big(\frac{1}{n_t^x}\big) + \frac{n_{t-1}-n_t^x}{n_{t-1}} \log\big(\frac{1}{n_{t-1}-n_t^x}\big)$$

$$= \log(n_{t-1}) - E[\mathrm{H}(P[M.|H_t])|s_t, r_t = x, H_{t-1}]$$

Hence, in order to show that

$$E[D_{\mathrm{KL}}(P[M.|H_t] \parallel P[M.|H_{t-1}])|s_t, r_t = d, H_{t-1}] \geq E[D_{\mathrm{KL}}(P[M.|H_t] \parallel P[M.|H_{t-1}])|s_t, r_t = f, H_{t-1}]$$

it is sufficient to show that

$$\log(n_{t-1}) - E[\mathrm{H}(P[M.|H_t])|s_t, r_t = d, H_{t-1}] \geq \log(n_{t-1}) - E[\mathrm{H}(P[M.|H_t])|s_t, r_t = f, H_{t-1}]$$

or equivalently, that

$$E[\mathrm{H}(P[M.|H_t])|s_t, r_t = d, H_{t-1}] \leq E[\mathrm{H}(P[M.|H_t])|s_t, r_t = f, H_{t-1}]$$

which we have already shown above.
